# Landscape heterogeneity affects diurnal raptor communities in a sub-tropical region of northwestern Himalayas, India

**DOI:** 10.1371/journal.pone.0246555

**Published:** 2022-04-28

**Authors:** Sudesh Kumar, Asha Sohil, Muzaffar A. Kichloo, Neeraj Sharma

**Affiliations:** 1 Department of Environmental Sciences, Govt. MAM College, Jammu, Jammu and Kashmir, India; 2 P.G. Department of Environmental Sciences, University of Jammu, Jammu, Jammu and Kashmir, India; 3 Department of Environmental Sciences, Govt. Degree College, Banihal, Jammu and Kashmir, India; 4 Institute of Mountain Environment, University of Jammu, Jammu, Jammu and Kashmir, India; Sikkim University, INDIA

## Abstract

Raptors are highly sensitive to environmental and human-induced changes. In addition, several species of raptors exist in considerably small numbers. It is thus critical to conserve raptors and their habitats across relatively larger landscapes. We examined the diurnal raptor assemblages and seasonality in a subtropical habitat in India’s northwestern Himalayas. Quantitative data on diurnal birds of prey and their habitat features across six distinct habitat types were collected from 33 sample sites. We observed 3,434 individuals of 28 diurnal raptors belonging to two orders and three families during a two-year survey from December 2016 to November 2018. A significant variation in bird species richness and abundance was found across habitats and seasons, with farmlands and winters being the most diverse and speciose. The generalized linear model, used to determine raptor community responses, indicated that elevation and proximity to dumping sites significantly affected the raptor abundance. The non-metric multidimensional scaling (NMDS) revealed significant differences in raptor assemblages across the habitat types. The study concluded that raptors’ persistence is largely determined by their preference for favourable feeding, roosting, and nesting opportunities. The presence of protected and habitat-exclusive species validates the high conservation importance of these ecosystems, particularly the forest patches and farmlands, necessitating robust conservation and management measures in this part of northwestern Himalaya.

## Introduction

With growing concerns about the changing land-use patterns, monitoring changes in the biological integrity of ecosystems has become essential [1], which may be accomplished using appropriate indicator species [2]. Raptors are considered excellent bio-indicators of habitat quality [3–5], environmental health [6, 7], and ecological imbalances [5, 8], and their existence is linked with a high level of biodiversity [4, 9]. Present in majority of ecosystems world over, although in very small numbers, they play a crucial role in organizing biological communities [10, 11] and promoting ecological stability [12]. However, a lack of information on the status, distribution, and ecological needs of raptors worldwide [13–15] hampers the conservation efforts [14, 16]. Raptors are particularly susceptible to human disruptions owing to their life-history characteristics, low population densities, large home ranges [17–19], and high trophic levels [4, 20]. Further, the environmental contamination [17] and habitat degradation [16, 21] exacerbate the rate of extinction [22], which is high among the raptors. Nonetheless, the adaptation of some raptors to human-altered environments places their candidature controversial as an indicator [23, 24].

Both high habitat heterogeneity and prey diversity contribute to species richness and abundance among the raptor communities [25], since species-environment interactions are largely determined by habitat types and their selection [26, 27]. Raptors require relatively large areas for effective hunting and nesting [28, 29], apart from avoiding human persecution. The understanding of biotic interactions, ecological affinities [30], and population dynamics of raptors provide valuable information about their habitats [7, 27, 31–35], enabling their optimal management and conservation [30, 36–38]. The avian diversity is linked to several environmental factors [39–41], most notably habitat [26] and seasonality, which determine the diversity and dynamics, including migrations in a variety of ecoregions [27, 35, 42–44]. Dietary preferences, habitat specializations, and migratory behavior significantly affect the distribution and richness of species across a vast geographical area [45].

Despite their charisma and immense ecological importance, there is no comprehensive global assessment of the status, threats, and protection of all raptors [46]. Their low population densities, slow turnover rates [6, 22, 47], and high susceptibility to anthropogenic stressors [4, 20] contribute to substantial population decline [48]. Other major limiting factors include habitat alteration [13, 14, 49–54], extermination [55, 56], poisoning [57–63], electrocution [64–67], collisions with man-made structures and vehicles [6, 68, 69], road kills [70], human consumption [71, 72], feral dog depredation [73], and climate change [74–79]. In addition, they are likely to become victims of expanding agriculture and logging globally [80, 81]. The majority of raptors, especially diurnal birds of prey, are the most vulnerable species [6], facing challenges throughout Europe [82, 83], Asia, the Middle East, and Africa [84–86]. Moreover, 127 of the world’s 333 species of diurnal birds of prey are found in Asia, 101 in the Indo-Malayan area, and 74 in India [87]. The erstwhile state of Jammu and Kashmir is home to 46 raptors [88], accounting for 62% of all birds of prey in India.

As much of the information comes from short-term studies [88–92] and opportunistic observations [93], data on functional traits and ecology of this largest avian group is scarce for the region [88]. Given the region’s relative lack of knowledge on raptor communities, we aimed to examine, how and to what degree their assemblages (distribution, abundance, and habitat associations) react to localized environmental characteristics and seasonality in various habitat types. During the two-year study, we predicted a pronounced variation in the raptor community structure (abundance, richness, composition) in open areas (with high chances of raptors sightings) and during winters (with the seasonal flocking by migrants). The study was conducted in Jammu *Shivaliks*, a southerly sub-tropical region in the Union Territory (UT) of Jammu and Kashmir.

## Material and methods

### Study area

**Fig 1:**
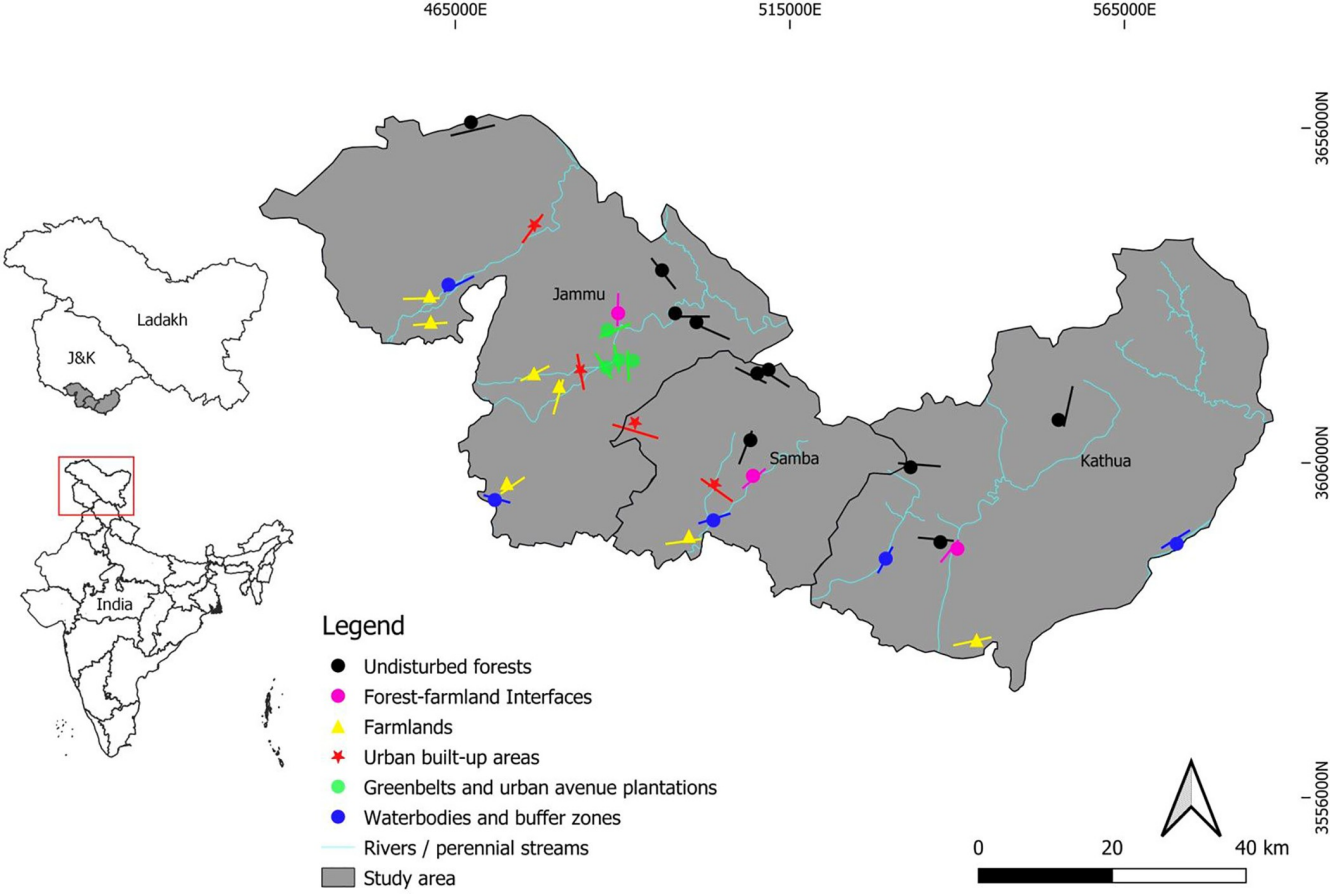
Location of sampling sites in the study area. The colored lines denote the sample clusters for each habitat type, which include road and line transects, while the symbols represent vantage points (refer S1 Appendix for details). All the spatial attributes were collected and prepared by the authors for the visualization purpose using open source QGIS; no copyrighted material was used.

The study area forms a complex heterogeneous land cover consisting of forests, fallow lands, agricultural fields, urban built-up areas, urban green spaces, and a variety of aquatic systems (S1 Appendix, Fig 1). Intended to cover a range of diverse habitats, the surveys were delimited to 33 sampling sites (micro-habitats) with varying degrees of disturbances (S1 Appendix). The sampling sites were classified as undisturbed forests (that included pristine mixed broadleaved forest, dry scrub, and riparian patches), forest farmland interfaces (edges between the forests and agriculture fields), farmlands (vast agricultural fields and fallow land), urban built-up areas (residential areas, including suburbs), green belts and urban avenue plantations (parks, gardens, roadside trees, remnant woodlands, plantations, and greenways), and water bodies and buffer zones that included seasonal and perennial ponds, streams, and rivers. The sampling was performed in four distinct seasons, summer (March–May), monsoon (June–September), post-monsoon (October–November), and winter (December–February) from December 2016 to November 2018.

### Topography and land use

The study area lies among the tertiaries and is comprised of two distinct geological formations, the *Shivaliks* and the alluvial plains, which serve as the northwestern extension of the Indo-Gangetic Plains. The *Shivalik* system, consisting of moderately elevated hills, is sedimentary in nature, dividing it into upper, middle, and lower zones. The region is drained by Chenab, Ravi, Tawi, Ujh, and Basanter as well as several seasonal streams locally known as *Khads*. The area is dotted with twin lakes, Surinsar and Mansar (both of which are Ramsar sites), reservoirs (Ranjitsagar and Ujh impoundment), and numerous wetlands, and seasonal and perennial ponds. Forests cover 38% of the geographical area, followed by agriculture (26%), open fallows (23%), grasslands (11%), and water bodies (2%).

### Sampling design and data collection

Diurnal raptors were recorded following the method described by Fuller and Mosher [94], Millsap and LeFranc [95], Whitcare and Turley [96], Austin et al. [97], and Bibby and Burgess [98]. We relied heavily on road surveys, which are the most effective methods for describing raptor assemblages, distribution, abundance, and habitat preferences [94, 99]. Each road transect spanned 3–7 kms and was traveled in a vehicle at a maximum speed of 20 km/h. The plains, farmlands, and undisturbed forests were sampled by setting up line transects and vantage points (point counts). The line transects (0.75–1.5 km each) were walked on foot at least twice a month during the entire sampling period. Each survey included teams of two to three observers. The vantage points were established in high locations to count flying raptors within a radius of 1–2 km. To prevent duplicate counting, the road and line transects were separated by at least 5–7 kms and 1 km, respectively. A total of 36 road transects, 19 line transects, and 80 vantage points were sampled in six different habitat types in the study area (S1 Appendix). For each transect, the number of species and individuals observed, activity, and habitats occupied were recorded. The sampling intensity, *i*.*e*., the number and length of transects, was justified in relation to the area occupied by diurnal raptors and their likelihood of occurrence. The surveys were performed in the mornings (9 a.m.–12 p.m.) and afternoons (3:30 p.m.–5:30 p.m.), which coincided with a time of high raptor activity [100]. Sampling was avoided during bad weather. A few opportunistic sightings near the specified sample sites were also included in the analysis. Observations were made using 10 × 50 binoculars and a Canon EoS 7D Mark II DSLR equipped with a 100 × 400 mm telephoto lens. The species observed were identified using standard field guides [101–103].

### Functional traits and conservation status

Raptors have been classified as predators (that hunt, kill, and eat their prey) or carrion feeders (that feed on the dead animal matter) based on their dietary choices and foraging behavior. For each site, the habitat guild (whether a generalist or specialist) and seasonal status, including whether it is a year-round resident, a summer or winter resident, for each species, was determined. The information on functional traits was extracted from De Graaf et al. [104] and The Cornell Lab of Ornithology [105]. The species’ conservation status was obtained from the IUCN Red List of Threatened Species [106].

### Data analysis

#### Richness and diversity attributes

Species diversity refers to the pooled number and summed abundance of each species in all months, seasons, habitat types, and the entire study area. It was determined using the Shannon–Weaver [107] and Simpson’s index [108], whereas species richness was the number of species per unit area [109]. The statistical analysis was performed using the Vegan library in the R programming environment [110].

#### Community responses to landscape-scale habitat factors

We used non-metric multidimensional scaling (NMDS) to assess bird community patterns in relation to landscape-scale habitat variables [111]. The analysis of similarity (ANOSIM) was performed to establish the significance of species composition across different habitat types [111]. The Simper analysis was used to assess the contribution of each species to community assemblages [111]. Species abundance was used to perform an ordination of habitat types in species space using the Bray–Curtis similarity index. A generalized linear model (GLM), with Poisson distribution and log link function in R, was used to assess the response of bird abundance to habitat variables. Bird abundance was used as the objective variable, whereas elevation, habitat features, and the distance to the closest dumping sites were used as explanatory variables. Elevation data were collected using GPS (Garmin-Montana 650), and the distance to the nearest sampling site was calculated using the distance matrix tool in QGIS [112]. We constructed two models of total abundance: one that contained both *Milvus migrans* and *M*. *m*. *lineatus*, and another that omitted both.

## Results

### Species richness and diversity

**Fig 2:**
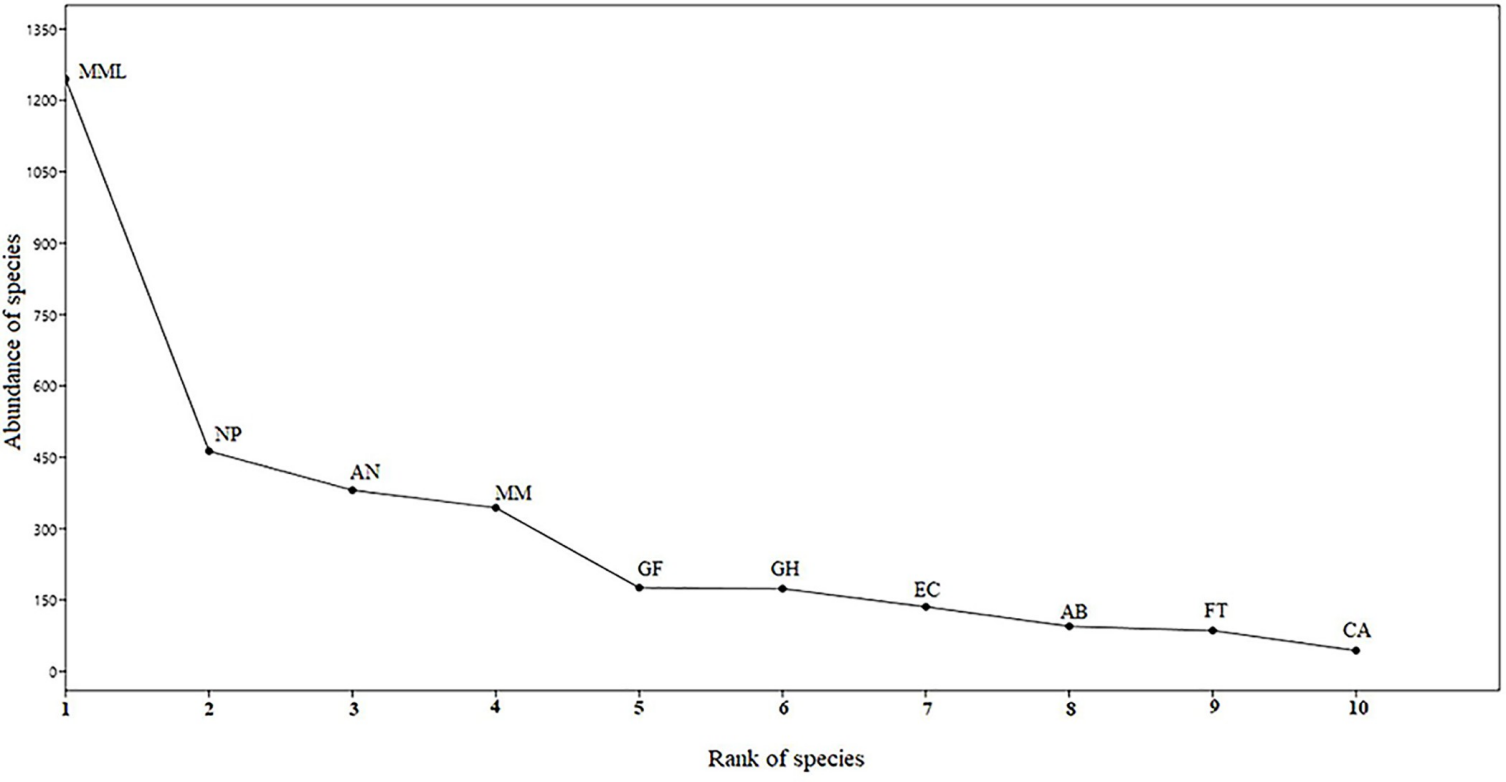
Rank abundance of ten dominant species / sub-species in the study area. MML: *Milvus migrans lineatus;* NP: *Neophron percnopterus;* AN: *Aquila nipalensis*, MM: *Milvus migrans*, GF: *Gyps fulvus*; GH: *Gyps himalayensis*; EC: *Elanus caeruleus*; AB: *Accipiter badius*; FT: *Falco tinnunculus*; CA: *Circus aeruginosus*.

In all, 3,434 individuals of 28 diurnal raptors belonging to two orders and three families were observed during sampling (S2 Appendix). Twenty four species belonged to the family Accipitridae, three to Falconidae and one to Pandionidae. The highest number of species was found in farmlands [23], followed by undisturbed forests [22], forest-farmland interfaces [20], and water bodies and buffer zones [19]. The habitats with fewer species included urban built-up areas, green belts, and urban avenue plantations (eight species each). A high mean abundance was found in urban built-up areas (34.68 ±21.44), followed by undisturbed forests (27.79 ±9.71), farmlands (18.49 ±4.91), forest farmland interfaces (14.45 ±6.18), water bodies (12.50 ±3.50), and green belts (11.48 ±7.59) (Table 1). *M*. *m*. *lineatus* had the highest relative abundance (RA = 36.28) followed by *Neophron percnopterus* (RA = 13.48) and *Aquila nipalensis* (RA = 10.75), whereas *Clanga clanga* and *Aquila rapax* had the lowest relative abundance and thus low ranking (S2 Appendix, Fig 2). Among the seasons, summers (41.03 ±16.51) recorded the highest mean abundance, followed by winters (32.37 ±11.17), monsoon (30.17 ±13.60), and post-monsoon (14.82 ±5.38) (Table 1). The diurnal raptor group as a whole exhibited a modest level of diversity (H′ = 3.50). Farmlands were the most diverse habitat type (H′ = 2.58), whereas urban areas were the least diverse (H′ = 1.14). Winters recorded the highest diversity and evenness (H′ = 2.42, J = 0.72), whereas monsoons had the least values (H′ = 2.01, J = 0.65) (Table 1). The species were more evenly dispersed in water bodies and buffer zones (J = 0.82) followed by farmlands (0.80) and undisturbed forests (0.70).

**Table 1:** Species richness and diversity of diurnal raptors in the study region as a function of habitats and seasons.

Habitat type / Season	Sampled area			Species richness and diversity attributes				
	Road transect	Line transect	Point count	Richness	Individuals / Km	Mean Abundance	Diversity (H′)	Evenness (J)
**Habitats**								
Undisturbed Forests	13*67 km	5*4.5 km	42	24	11.27	27.79 ±9.71	2.25	0.70
Forest Farmland interfaces	2*11 km	1*1.5 km	5	22	33.52	14.45 ±6.18	2.08	0.67
Farmlands	8*32 km	4*4.0 km	11	25	14.50	18.49 ±4.91	2.58	0.80
Urban built-up areas	3*17 km	1*1.5 km	4	8	54.37	34.68 ±21.44	1.14	0.55
Green belt and avenue plantations	3*9 km	4*2.0 km	2	8	30.27	11.48 ±7.59	1.18	0.56
Water bodies and buffer zones	6*33 km	3*4.5 km	16	21	9.28	12.50 ±3.50	2.50	0.82
**Seasons**								
Winter	35*169 km	18*18 km	80	29	5.02	32.37 ±11.17	2.42	0.72
Summer				26	6.36	41.03 ±16.51	2.11	0.64
Monsoon				22	4.67	30.17 ±13.60	2.01	0.65
Post-Monsoon				27	2.30	14.82 ±5.38	2.30	0.70

In terms of habitat usage, 15 birds (53%) of all observed species were specialists, whereas 13 were generalists. Twelve of the specialists were forest raptors (*n* = 576), whereas 3 were water-dependent (*n* = 51). Observations on the food type and foraging behavior identified two broad trophic guilds, namely predators (22 species, *n* = 998) and carrion feeders (6 species, *n* = 2,436). Eight species of forest specialists were predators, whereas the remaining (all vultures) were carrion feeders. Eleven among the generalists were predators, whereas the remaining two were carrion feeders (Table 2).

**Table 2:** Habitat and forging guilds of diurnal raptors in the study area.

**Habitat Guilds**					
**Generalists**		**Specialists**			
		**Forest**		**Aquatic / dependent**	
*Neophron percnopterus*, *Milvus migrans*, *M*. *m*. *lineatus Aquila nipalensis*, *A*. *heliaca*, *A*. *rapax Elanus caeruleus*, *Accipiter badius*, *Buteo buteo*, *B*. *rufinus*, *Circaetus gallicus*, *Clanga hastata*, *C*. *clanga*, *Falco peregrinus*		*Pernis ptilorhynchus*, *Falco subbuteo*, *F*. *tinnunculus*, *Accipiter nisus*, *Aquila fasciata*, *Butastur teesa*, *Gyps fulvus*, *G*. *himalayensis*, *G*. *bengalensis*, *Hieraaetus pennatus*, *Spilornis cheela*, *Aegypius monachus*		*Pandion haliaetus*, *Circus cyaneus*, *C*. *aeruginosus*	
**Feeding Guilds**					
**Predator**	**Carrion feeder**	**Predator**	**Carrion feeder**	**Predator**	**Carrion feeder**
11	2	8	4	3	-

### Species similarity and habitat exclusiveness

**Fig 3:**
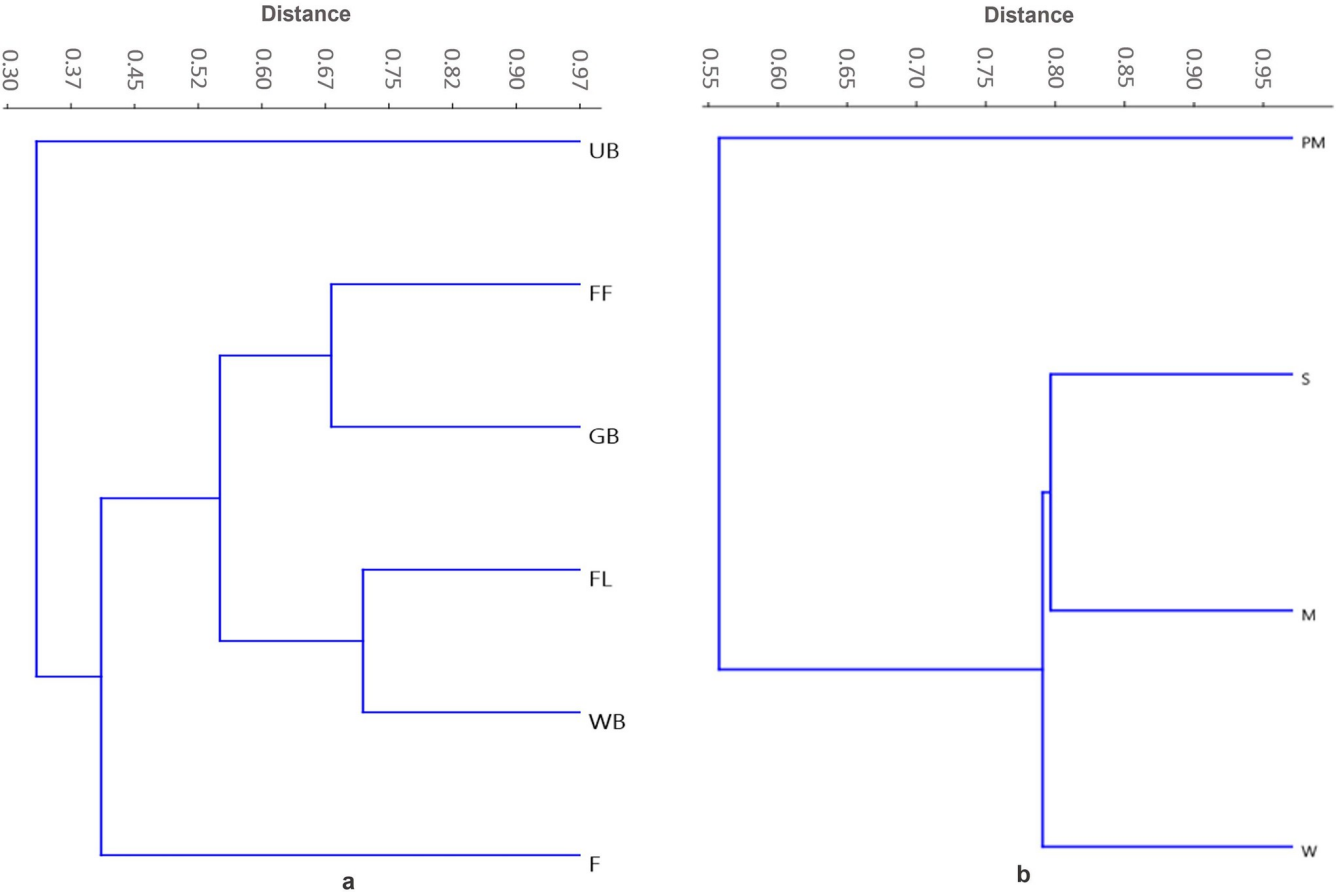
Abundance based Bray-curtis similarity linkages between (a) habitat types (b) seasons. FR: Undisturbed forests; FF: Forest farmland interfaces; FL: Farmlands; UB: Urban built-up areas; GB: Green belt and urban avenue plantations; WB: water bodies and buffer zones; PM: Post monsoon; S: Summer; M: Monsoon; W: Winter.

*Milvus migrans*, *M*. *m*. *lineatus*, *Aquila nipalensis*, *A*. *badius*, *Hieraaetus pennatus*, *Falco peregrinus*, and *Neophron percnopterus* were found in all habitat categories and were considered ubiquitous. Among the habitats, farmlands shared a maximum of 21 species with undisturbed forests, followed by forest farmland interfaces and water bodies, which shared 20 species. Similarly, undisturbed forests shared 20 species with forest farmlands interfaces, followed by water bodies and buffer zones (17 species). Winters shared 27 species with the post-monsoon season, which was followed by summers (26 species). Summer and post-monsoon shared the maximum (25 species). Bray–Curtis similarity linkages across habitat types showed the formation of four clusters, namely forest farmland interfaces-water bodies and farmlands-green belt parks that had comparable raptor assemblages. The third and fourth clusters included densely populated urban areas and undisturbed forests with distinct raptor associations (Fig 3A). Among the seasons, summer, winter, and monsoon had comparable raptor assemblages and were therefore grouped, whereas the post-monsoon assemblages remained distinct and formed a separate cluster (Fig 3B).

### Whittaker curves

**Fig 4:**
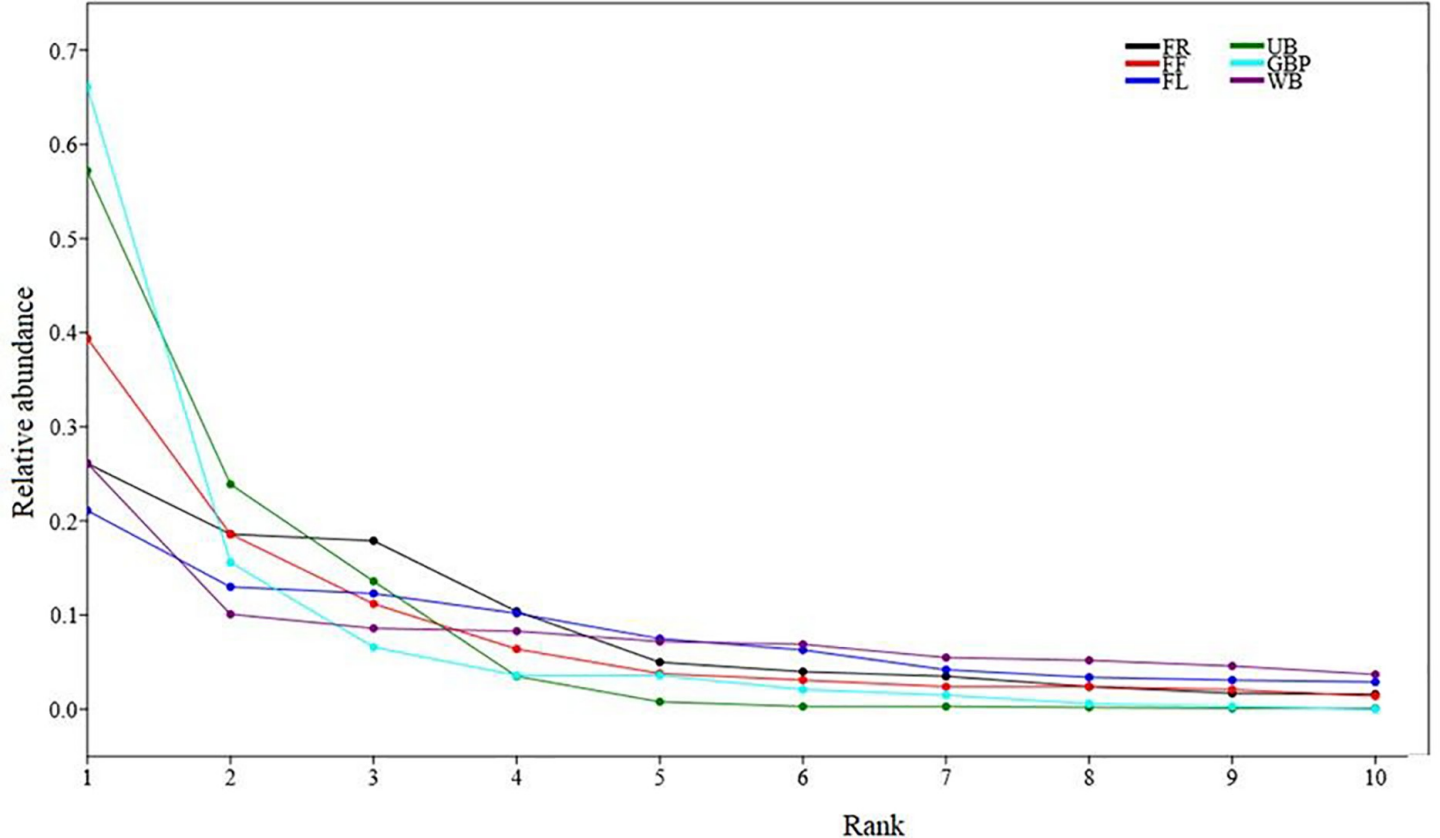
Rank abundance (Whittaker curves) of top ten diurnal raptors. Coloured lines represent rank abundance diversity curves for six habitat types.

Whittaker curves (Fig 4) showed the species diversity among habitat categories, with green belts and urban built-up areas ranking the highest in terms of raptor abundance, followed by undisturbed forests and forest farmland interfaces. Water bodies and farmlands received a low ranking. Undisturbed forests, water bodies, farmlands, and forest farmland interfaces with slanting curves exhibited a high degree of species richness and evenness, whereas urban built-up areas and green belts were less speciose.

### Community responses to landscape-scale habitat factors

**Fig 5:**
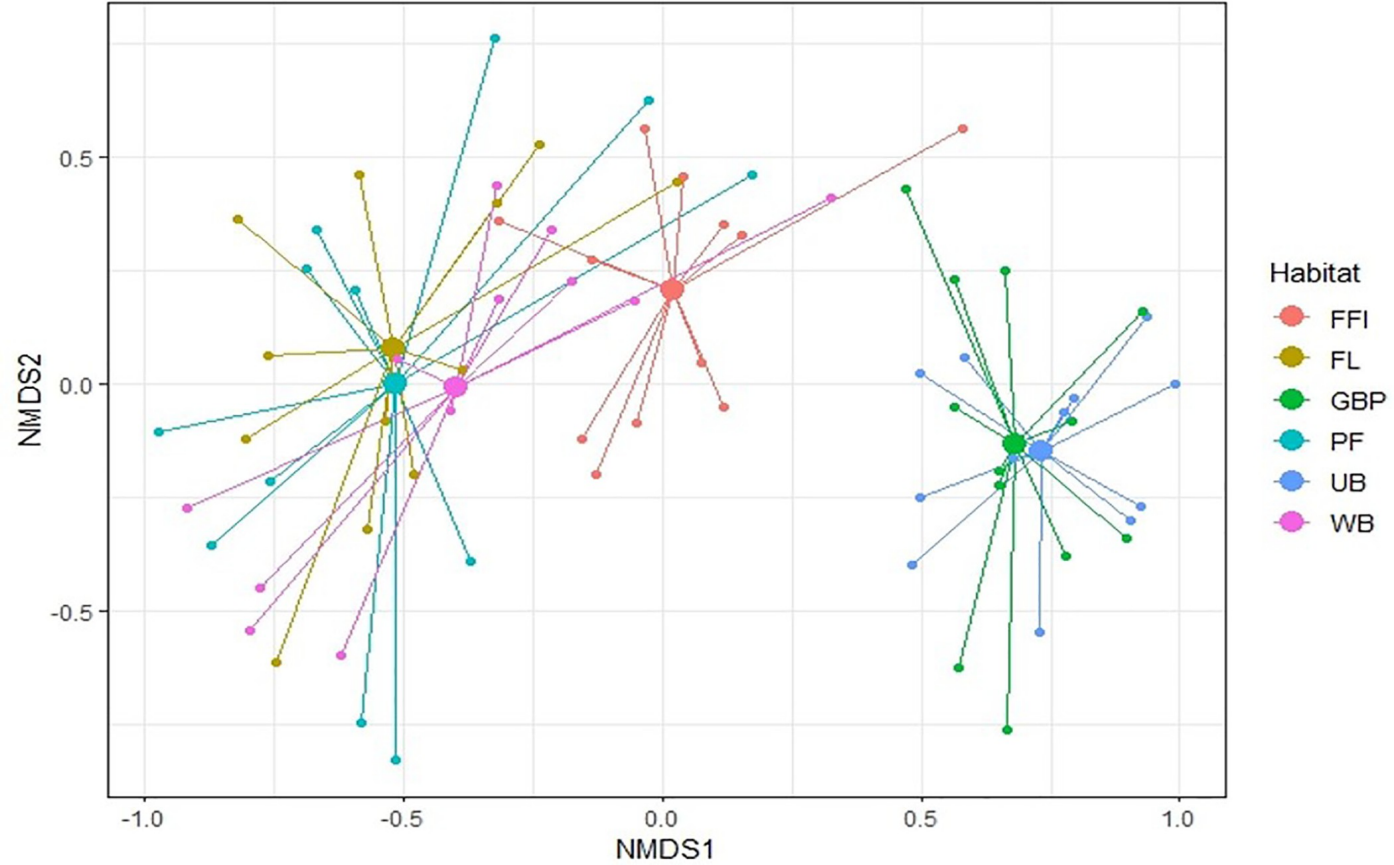
Bird species composition and community patterns among landscape-scale habitat factors. Non-metric multidimensional scaling (NMDS) plot depicting raptor community assemblages in six contrasting habitat types using Bray-Curtis similarity. Pair wise ANOSIM tests revealed significant variation (p < 0.05) in raptor compositions. The vectors that stretch up to the point denote species, whereas the six big dots indicate habitat categories.

Raptor assemblages constructed using NMDS (Fig 5) showed a significant variation in species composition across habitats (ANOSIM, number of permutations = 999; global R = 0.63; *p* = 0.0001), with observed species dissimilarity of 63.26% (R^2^ = 0.17). The SIMPER-based average species dissimilarity between forested sites (undisturbed forest and forest-farmland interfaces) was 67.02%. It consisted of 11 species, which accounted for more than 90% of the overall species composition. Table 3 summarizes the contribution of representative species and the average dissimilarity for different habitat categories.

**Table 3:** SIMPER results explaining contribution percentage (similarity) of representative species / sub-species and average dissimilarity for the habitat types.

Study Site	Average dissimilarity	Species	Contribution %	Cumulative %
Forests (undisturbed forests, forest	67.22%	*Aquila nipalensis*	15.32	15.32
farmland interfaces)		*Accipiter badius*	13.61	28.93
		*Falco subbuteo*	12.46	41.39
		*Gyps himalayensis*	11.97	53.37
		*Gyps fulvus*	11.75	65.11
		*Milvus migrans lineatus*	9.96	75.07
		*Neophron percnopterus*	5.52	80.59
		*Milvus migrans*	5.16	85.75
		*Elanus caeruleus*	2.31	88.06
		*Spilornis cheela*	1.64	89.70
		*Circaetus gallicus*	1.30	91.00
		*Accipiter badius*	35.00	35.00
Farmlands (forest farmland	57.02%	*Milvus migrans lineatus*	8.95	43.95
interfaces / farmlands)		*Circus aeruginosus*	8.77	52.72
		*Aquila nipalensis*	8.09	60.81
		*Elanus caeruleus*	6.88	67.68
		*Neophron percnopterus*	5.89	73.57
		*Milvus migrans*	5.71	79.28
		*Falco tinnunculus*	3.96	83.24
		*Clanga hastata*	2.10	85.34
		*Buteo rufinus*	2.09	87.43
		*Butastur teesa*	2.04	89.46
		*Gyps himalayensis*	1.47	90.93
Urban areas (Urban built-up areas, Green	53.98%	*Milvus migrans lineatus*	47.92	47.92
belts and urban avenue plantations)		*Neophron percnopterus*	30.76	78.68
		*Milvus migrans*	12.78	91.46
Water bodies (seasonal and perennial	73.51%	*Milvus migrans lineatus*	46.52	46.52
ponds, streams and rivers)		*Neophron percnopterus*	21.45	67.98
		*Milvus migrans*	11.04	79.02
		*Aquila nipalensis*	4.00	83.02
		*Circus aeruginosus*	2.48	85.50
		*Falco tinnunculus*	1.95	87.45
		*Gyps himalayensis*	1.91	89.37
		*Accipiter badius*	91.27	2.08

### Effect of habitat characteristics on bird species abundance

The avian species abundance responded differently to the contrasting habitats when tested using the GLM (Table 4). In model one, which included the urban commensals *M*. *migrans* and *M*. *m*. *lineatus*, the abundance of all raptors was governed by elevation and distance to the nearest dumping sites. Except for water bodies and buffer zones, the abundance of species in all other habitat types differed significantly from farmlands. The gregarious assemblages of *M*. *migrans* and *M*. *m*. *lineatus* drive a high bird abundance in urban built-up areas. In the second model that excluded the commensals, the abundance of bird species was more strongly linked to elevation. The abundance decreased and remained low for forest farmland interfaces and urban built-up areas. Greenbelts and urban avenue plantations were expected to have the lowest abundance, implying their unsuitability for non-commensal raptors. In contrast, undisturbed forests and water bodies were predicted to behave better than greenbelts and urban avenue plantations. Non-commensal raptors preferred the forest-farmland interface and urban built-up regions.

**Table 4:** Generalized linear model (GLM) explaining relationship between raptor abundance and habitat characteristics. Two models, one that included the commensals *M*. *migrans* and *M*. *m*. *lineatus* and second, excluding them, were run to analyze effect of habitat variables on raptor abundance.

Model	Variables	Estimate	Std. error	Z value	P value
**Abundance (Including Commensals)**	Intercept	4.215	0.062	67.263	0.000***
	Elevation	0.745	0.080	9.219	0.000***
	Distance to nearest dumping sites	-0.367	0.037	-9.894	0.000***
	Forest Farmland Interfaces	0.398	0.069	5.754	0.000***
	Undisturbed forests	-0.551	0.091	-6.018	0.000***
	Green belts and urban avenue plantation	-0.289	0.079	-3.665	0.000***
	Urban built-up areas	0.923	0.060	15.239	0.000***
	Water bodies and buffer zones	-0.135	0.070	-1.921	0.054˚
**Abundance (Excluding Commensals)**	Intercept	3.189	0.072	53.02	0.000***
	Elevation	0.829	0.091	9.10	0.000***
	Distance to nearest dumping sites	-0.367	0.044	-8.21	0.000***
	Forest farmland Interfaces	0.022	0.090	0.24	0.803
	Undisturbed Forests	-0.433	0.105	-4.12	0.000***
	Green belts and urban avenue plantation	-1.646	0.143	-11.45	0.000***
	Urban built-up areas	0.035	0.084	0.14	0.674
	Water bodies and buffer zones	-0.258	0.086	-2.98	0.002**

P value represents significance codes: 0

‘***’; 0.001

‘**’; 0.01

‘*’; 0.05 ‘˚’.

### Conservation status

Nine species, *Aquila nipalensis*, *A*. *heliaca*, *A*. *rapax*, *Circus cyaneus*, *Buteo rufinus*, *B*. *buteo*, *Clanga clanga*, *Aegypius monachus* and *Pandion haliaetus* were winter visitors. *Falco subbuteo* was found to be summer visitor whereas the remaining 18 were residents. Nine species were recognized as globally threatened (IUCN, 2020), which included *Gyps bengalensis* (critically endangered), *Neophron percnopterus* and *Aquila nipalensis* (endangered), *Clanga hastata*, *C*. *clanga*, *Aquila heliaca* and *A*. *rapax* (vulnerable), *Gyps himalayensis* and *Aquila monachus* (near threatened). The least concern group included 19 species (S2 Appendix).

## Discussion

Twenty-eight species, reported during the present study accounted for 38% of all diurnal raptors in India [87] and 62% of those in the erstwhile state of Jammu and Kashmir [88]. Statistical analysis revealed that different habitat types differed in terms of species richness and abundance. Undisturbed forest patches, rocky cliffs and ridges, vast fallows and agricultural fields, water bodies (rivers, streams, and ponds), floodplains, and urban habitats infused with green spaces provide a favorable space for nesting, breeding, perching, and roosting, thereby resulting in a high raptor richness and abundance [17, 113–116]. Significant variation in species richness and abundance among habitat types was probably related to the morphology, hunting tactics, nesting, and foraging requirements [117], habitat condition, migratory behavior, and breeding season of the species [118, 119], in addition to human disturbances [120]. Landscape attributes determine avian richness and abundance [121, 122], which is high in mosaic lands [123–125] and limited by suitable breeding habitat and specific nest-site requirements [17, 84].

GLM results suggested that the abundance of raptors was regulated by elevation, habitat use, and distance to the dumping sites. Differential habitat usage, large home range [126], and elevational gradient could be the key drivers contributing to high raptor abundance. The higher elevations occupied by sub-tropical broad-leaved forests interspersed with Chirpine, with little human disturbance could be another reason for high raptor diversity. In addition, this high number could be linked to a stable and abundant prey base, low competition, and adequate nesting provisions [127]. *Milvus migrans* and *M*. *m*. *lineatus*, the most abundant urban commensals, accounted for more than 40% of the total observed raptor population. These generalists use man-made structures (building, bridges, towers, poles), urban green spaces, and stormwater drains as shelter, nesting sites, and food sources (including offal and anthropogenic refuse), achieving the largest numbers in urban localities [128]. Raptors are drawn to urban environments by perching locations near roadways (power lines and telephone poles) and road kills [129]. Numerous studies have demonstrated that urbanization impacts the diversity, composition, and abundance of bird communities [130–132], confirming the increased abundance of urban commensals in the study area. Because urbanization leads to biological homogeneity, urban-adapted species have become more common and locally abundant [133]. Medium-sized raptors successfully inhabit these habitats [134, 135] by locating ideal food, breeding, and roosting locations [136]. However, urbanization exerts a detrimental effect on vulnerable species [137, 138]. Eagles, hawks, and falcons [139] with unique habitat requirements [128] are more abundant in less disturbed and natural habitats [1, 128, 140] that provide a safe refuge, hostile environment, and prey species [126]. It consists of forest specialists, migrants, and nesting birds that are particularly sensitive to human presence.

The richness and diversity attributes increased with increased habitat heterogeneity. Agricultural areas and wide-open spaces serve as nesting and foraging sites for a variety of open space foragers, including buzzards and harriers [12, 141–144]. Apart from creating new habitats, irrigated crops increase the availability of food for birds of prey in the form of small mammals, voles, and rodents, which are ideal prey for western marsh harriers [145, 146], black kites [147, 148], black-winged kites, and migrant raptors such as booted eagle and steppe eagle [31, 149, 150]. Our study demonstrates the critical nature of forests and farmlands, which are home to over 90% of all raptor species recorded in the study area. Forests, as raptor habitats, are more vulnerable and hence demand conservation and effective protection [15, 151, 152]. Seven species, mostly residents, were identified across all landscape types being resilient to or having acclimated to landscape changes throughout time [12].

Given that food availability is a significant determinant of raptor density [17], the distance from dumping sites reduces human-subsidized food availability, thereby negatively affecting the raptor population. This supports the GLM results reported in the current study. Apart from birds and mammals [126], reptiles, amphibians, fish, and arthropods are the primary dietary sources for raptors [153]. Twenty-two species were recognized as predators and 6 as carrion feeders based on foraging data. *M*. *migrans*, *M*. *m*. *lineatus*, and *N*. *percnopterus* were the top most prevalent species along urban roadways, water bodies, and perches and found either foraging or roosting.

Others, such as *G*. *fulvus*, *G*. *himalayensis*, *G*. *bengalensis*, and *A*. *monachus*, were found in the undisturbed forests and/or forest-farmland interfaces, where they fed on dead wildlife and livestock. Carrion from large animals, deer, and hares in the forest may provide a rich food source for raptors [126, 154]. Unlike scavenging raptors, predatory raptors visually search for and hunt their prey [155]. The observed richness of predatory raptors was significantly higher than that of scavengers, which could be explained by the abundance of different food types in the study area’s mosaic ecosystems. Food availability [156] is the most important criterion for selecting suitable stopover sites for wintering [157]. Predatory winter migrants, including resident raptors, were frequently spotted feeding on lizards, rodents, insects, and birds in mosaic environments. Raptor size and diet appeared to be the most promising characteristics defining birds’ extensive patterns [54].

Although raptor species occupied a variety of habitats [158], no seasonal variation in raptor abundance was detected. However, despite the absence of a seasonal pattern, monthly changes in species richness and abundance were observed [159]. Ten species were migrants, including 9 winter visitors and 1 summer visitor, compared to 18 resident species. Seasonal migration patterns, local and regional habitat changes, large-scale population fluctuations, and climatic conditions may contribute to this migratory behavior [160–162]. During the study period, most of the winter migrants were reported from paddy fields and other farmlands located near wetlands or open areas close to streams and floodplains. During winters, only a few were observed in the undisturbed forests. Migrating species frequently use paddy fields as foraging habitats throughout the winter due to the presence of snakes, rodents, and crustaceans [163, 164]. In addition, the forest serves as a refuge for migratory species as a place to rest and feed until more favorable conditions return [165, 166]. Interestingly, five of the nine globally threatened raptors [106] were migrants, rendering them more vulnerable to threats [52, 167, 168], including the migration-related mortality [169]. The presence of globally threatened species, with a predominance of migratory raptors in the study area, substantiates its designation as a region’s top raptor conservation priority area.

Dissimilarity in community composition is one of the conspicuous features of community ecology [170, 171]. Comparing the compositions of different habitat types revealed the key mechanisms and habitat-specific impacts that shaped biodiversity composition and structure [172], which is critical for analyzing species invasions, changes induced by habitat fragmentation, and the effects of climate change. The average dissimilarity in species composition between habitat types was 63.26%, which could be attributed to the heterogeneous nature of habitats containing several ecosystems and exhibiting a range of environmental traits that contribute to community composition and variety.

## Conclusions

Our study emphasizes the critical role of urban, forested, agricultural, and aquatic environments in the monitoring and conservation of raptors. A combination of natural, semi-natural, and urban environments serves as hotspots of landscape diversity, allowing the coexistence of a diverse range of species with varying habitat requirements. Numerous migratory species, including a few globally threatened birds, reflect the habitat’s uniqueness and potentiality. Effective strategies are required to improve, protect, and conserve these ecosystems to sustain biological integrity, avoid species extinction, and accelerate species recovery. We believe this study has significant implications for future efforts to conserve raptors, particularly in this region of the northwestern Himalayas.

## Supporting information

S1 Appendix Spatial attributes of sample locations, including geomorphological features, sampling plots, and the degree of disturbance. (DOCX)

S2 Appendix List of diurnal raptors recorded in the study area, their abundance, guilds and conservation status. (DOCX)

S1 Table (XLSX)

S1 File (DOCX)
