# Supplementary material for: Landscape heterogeneity affects diurnal raptor communities in a sub-tropical region of northwestern Himalayas, India
Source: PLoS One. 2022 Apr 28;17(4):e0246555. doi: 10.1371/journal.pone.0246555 (PMC9049523; doi:10.1371/journal.pone.0246555)
Supplement: S1 Appendix — (DOCX) [file pone.0246555.s001.docx]

**S1 Appendix : Spatial attributes of sample locations, including geomorphological features, sampling plots, and the degree of disturbance**

| **Study Site /**  **Habitat** | **Transect type / No** | **Sampling sites** | **Geo-Coordinates** | **Elev.**  **(in m)** | **Habitat types** | **Disturbance** |
| --- | --- | --- | --- | --- | --- | --- |
| Undisturbed Forests | Road=13 | Kalidhar | 33.0500N, 74.6500E | 845 | Dominated by Chirpine-broadleaved mixed forests | Low |
|  | Line=5 | Billawar | 32.6480N, 75.5880E | 1176 | Sub-tropical broadleaved forests dominated with Chirpine at hill tops | Moderate |
|  | Point=42 | Galak | 32.5850N, 75.3520E | 610 | Rocky outcrops interspersed with open grassy slopes and Chirpine forests. | Moderate |
|  |  | Nandini | 32.8510N, 74.9560E | 610 | Area characterized with sub-tropical deciduous forests interspersed with Chirpine. | Low |
|  |  | Jasrota | 32.4840N, 75.3990E | 560 | Sub-tropical dry deciduous forest interspersed with thick bamboo stands | Low |
|  |  | Sarain | 32.6220N, 75.0970E | 475 | Sub-tropical dry open scub | High |
|  |  | Bhed devta | 32.7930N, 74.9770E | 447 | River bed and riparian forest | Moderate |
|  |  | Badgah | 32.7810N, 75.0110E | 632 | Lake, agriculture and human habitations | Low |
|  |  | Battal | 32.7170N, 75.1260E | 674 | Lake, agriculture and commercial setups | Low |
|  |  | Kumbi | 32.7120N, 75.1080E | 772 | Plateau with sparse Chiprine stands overlooking deep ravines and gorges | Moderate |
| Forest-farmland  Interfaces | Road=2 | Ujh Barrage | 32.4750N, 75.4260E | 386 | A lacustrine ecosystem surrounded with plantations, farmlands and habitations. | Moderate |
|  | Line=1 | Nagrota | 32.7930N, 74.8860E | 430 | Undulating valleys with flat sub-tropical hills | High |
|  | Point=5 | Samba | 32.5740N, 75.1010E | 354 | Subtropical dry scrub and vast agriculture landscapes southwards | High |
| Farmlands | Road=8 | Jourian | 32.8160N, 74.5850E | 268 | Vast cultivable farmlands | Low |
|  | Line=4 | Pargwal | 32.7990N, 75.5950E | 263 | Waterlogged fields and fallows | Low |
|  | Point=11 | Kokerian | 32.7120N, 74.7520E | 265 | A wide and shallow channel of river Tawi | High |
|  |  | Khakryal | 32.3520N, 75.4560E | 289 | Vast arable agriculture landscapes | Low |
|  |  | Phallian Mandal | 32.6950N, 74.7920E | 275 | Low lying farmlands and fallow lands | High |
|  |  | Koulpur | 32.4930N, 74.9990E | 303 | Vast open rice fields | Low |
|  |  | Gharana cultivation fields | 32.5410N, 74.6900E | 263 | agriculture landscapes dotted with habitations around Gharana wetland conservation reserve | Moderate |
| Urban built-up | Road=4 | Nikki Tawi | 32.7160N, 74.8260E | 289 | A natural off shoot of river Tawi southwards of Jammu City, with moderate pollution load | Very high |
|  | Line=1 | Bari Brahmana | 32.6460N, 74.9130E | 332 | Industrial area surrounded by vast wastelands and fallows | High |
|  | Point=4 | Akhnoor | 32.912N, 74.752E | 304 | A township located along the banks of river Chenab. | High |
|  |  | Vijaypur | 32.5630N, 75.0400E | 340 | Sub-urban area along the right bank of seasonal Devak stream | High |
| Green belt and urban avenue plantations | Road=3 | Raika | 32.7290N, 74.9100E | 515 | A raised forest with mix of native and introduced trees. Understory comprises of native shrubs | Moderate |
|  | Line=5 | Mahamaya | 32.7300N, 74.8870E | 402 | A natural remnant of forest, a designated protected area | Low |
|  | Point=2 | Manda | 32.7700N, 74.8690E | 551 | Urban patch bordering Ramnagar Wildlife Sanctuary northwards of Jammu city. | Low |
|  |  | Jammu University | 32.7200N, 74.8670E | 317 | A green residential campus sprawled in 118 acres on mid of Jammu city along the left banks of river Tawi | Low |
| Water bodies and buffer zones | Road=6 | Daya chak | 32.4620N, 75.3120E | 362 | Vast landmass dotted with water bodies | High |
|  | Line=3 | Basantar | 32.5140N, 75.0380E | 313 | A perennial medium stream with sandy bed drains Samba and adjoining villages | Moderate |
|  | Point=16 | Jourian | 32.8160N, 74.5850E | 268 | Vast landmass of irrigated agricultural land | Low |
|  |  | Gharana | 32.5410N, 74.6900E | 263 | A designated protected wetland and important Bird Area, a heaven for winter migrants. | Moderate |
|  |  | Purthu | 32.4800N, 75.7750E | 525 | South-western part of vast Ranjit Sagar Reservoir in district Kathua | Low |
