# Supplementary material for: Landscape heterogeneity affects diurnal raptor communities in a sub-tropical region of northwestern Himalayas, India
Source: PLoS One. 2022 Apr 28;17(4):e0246555. doi: 10.1371/journal.pone.0246555 (PMC9049523; doi:10.1371/journal.pone.0246555)
Supplement: S2 Appendix — (DOCX) [file pone.0246555.s002.docx]

**S2 Appendix: List of diurnal raptors recorded in the study area, their abundance, guilds and conservation status**

| **S. No.** | **Order/ Family/ Common name** | **Binomial name** | | **Relative Abundance (RA)** | | | | | | **RA**  **( % )** | **MS** |  |  |  |
| --- | --- | --- | --- | --- | --- | --- | --- | --- | --- | --- | --- | --- | --- | --- |
|  |  |  |  | **Winter** | **Summer** | | **Monsoon** | | **Post monsoon** |  |  | **Feeding**  **Behaviour** | **Habitat Guild** | **IUCN** |
| **ORDER: ACCIPITRIFORMES** | | | | | | | | | | | | | | |
| **Family: Pandionidae** | | | | | | | | | | | | | | |
| 1 | Osprey | *Pandion haliaetus* | | 0.003 | 0.001 | | 0.000 | | 0.002 | 0.15 | W | Predator | Obligate Wetland Specialist  (WB) | LC |
| **Family: Accipitridae** | | | | | | | | | | | | | | |
| 2 | Black-winged kite | *Elanus caeruleus* | 0.04 | | 0.03 | 0.05 | | 0.04 | | 3.96 | R | Predator | Generalist  (FF, FL, GP) | LC |
| 3 | Egyptian Vulture | *Neophron percnopterus* | 0.11 | | 0.14 | 0.15 | | 0.15 | | 13.48 | R | Carrion eater | Generalist  (UB, FF, FL, GP, WB) | EN |
| 4 | Oriental honey-buzzard | *Pernis ptilorhynchus* | 0.01 | | 0.01 | 0.01 | | 0.01 | | 0.93 | R | Predator | Forest Specialist  (FR, FF, GP) | LC |
| 5 | Cinereous vulture | *Aegypius monachus* | 0.01 | | 0.002 | 0.00 | | 0.004 | | 0.32 | W | Carrion eater | Forest Specialist  (FL, FF) | NT |
| 6 | White-rumped Vulture | *Gyps bengalensis* | 0.01 | | 0.002 | 0.004 | | 0.01 | | 0.47 | R | Carrion eater | Forest Specialist  (FF) | CR |
| 7 | Himalayan Vulture | *Gyps himalayensis* | 0.06 | | 0.06 | 0.04 | | 0.02 | | 5.07 | R | Carrion eater | Forest Specialist  (FR, FF) | NT |
| 8 | Griffon Vulture | *Gyps fulvus* | 0.04 | | 0.06 | 0.05 | | 0.05 | | 5.21 | R | Carrion eater | Forest Specialist  (FR, FF) | LC |
| 9 | Crested Serpent Eagle | *Spilornis cheela* | 0.001 | | 0.006 | 0.002 | | 0.01 | | 0.47 | R | Predator | Forest Specialist  (FR) | LC |
| 10 | Short-toed Snake eagle | *Circaetus gallicus* | 0.01 | | 0.003 | 0.01 | | 0.01 | | 0.84 | R | Predator | Generalist  (FL, WB, FR) | LC |
| 11 | Indian Spotted Eagle | *Clanga hastata* | 0.01 | | 0.003 | 0.002 | | 0.01 | | 0.67 | R | Predator | Generalist  (FM, WB) | VU |
| 12 | Greater Spotted Eagle | *Clanga clanga* | 0.003 | | 0.000 | 0.00 | | 0.002 | | 0.12 | W | Predator | Generalist  (FF, FL) | VU |
| 13 | Booted Eagle | *Hieraaetus pennatus* | 0.01 | | 0.005 | 0.001 | | 0.01 | | 0.55 | R | Predator | Generalist  (WB, FL, UB) | LC |
| 14 | Tawny Eagle | *Aquila rapax* | 0.003 | | 0.000 | 0.00 | | 0.000 | | 0.09 | W | Predator | Generalist  (FL, WB) | VU |
| 15 | Steppe Eagle | *Aquila nipalensis* | 0.13 | | 0.14 | 0.07 | | 0.05 | | 10.75 | W | Predator | Generalist  (FL, WB) | EN |
| 16 | Eastern Imperial Eagle | *Aquila heliaca* | 0.01 | | 0.003 | 0.00 | | 0.004 | | 0.41 | W | Predator | Generalist  (FL) | VU |
| 17 | Bonelli's Eagle | *Aquila fasciata* | 0.004 | | 0.001 | 0.00 | | 0.000 | | 0.15 | R | Predator | Forest Specialist  (FR, FL) | LC |
| 18 | White-eyed Buzzard | *Butastur teesa* | 0.01 | | 0.01 | 0.01 | | 0.01 | | 1.08 | R | Predator | Forest Specialist  (FR, FF) | LC |
| 19 | Eurasian Marsh Harrier | *Circus aeruginosus* | 0.01 | | 0.01 | 0.01 | | 0.01 | | 1.16 | R | Predator | Facultative wetland Specialist  (WB, FL) | LC |
| 20 | Hen Harrier | *Circus cyaneus* | 0.004 | | 0.000 | 0.00 | | 0.004 | | 0.17 | W | Predator | Facultative wetland Specialist  (WB, FL) | LC |
| 21 | Shikra | *Accipiter badius* | 0.03 | | 0.03 | 0.03 | | 0.03 | | 2.94 | R | Predator | Generalist  (FR, FF, FL, UB, GP, WB) | LC |
| 22 | Eurasian Sparrowhawk | *Accipiter nisus* | 0.002 | | 0.001 | 0.001 | | 0.004 | | 0.17 | R | Predator | Forest Specialist  (FR, FL) | LC |
| 23 | Black Kite  Black-eared Kite | *Milvus migrans govinda*  *Milvus migrans lineatus* | 0.09  0.32 | | 0.09  0.37 | 0.08  0.43 | | 0.19  0.30 | | 10.10  36.28 | R | Carrion eater | Generalist  (FR, FF, FL, UB, GP, WB) | LC |
| 24 | Common Buzzard | *Buteo buteo* | 0.01 | | 0.001 | 0.002 | | 0.002 | | 0.35 | W | Predator | Generalist  (FL, FF, WB) | LC |
| 25 | Long-legged Buzzard | *Buteo rufinus* | 0.02 | | 0.01 | 0.01 | | 0.01 | | 1.08 | W | Predator | Generalist  (FR, FL, WB, FF) | LC |
| **ORDER: FALCONIFORMES** | | | | | | | | | | | | | | |
| **Family: Falconidae** | | | | | | | | | | | | | | |
| 26 | Common Kestrel | *Falco tinnunculus* | 0.02 | | 0.02 | 0.02 | | 0.03 | | 2.07 | R | Predator | Forest Specialist  (FR, FF, FL, WB) | LC |
| 27 | Eurasian Hobby | *Falco subbuteo* | 0.001 | | 0.004 | 0.003 | | 0.002 | | 0.29 | S | Predator | Forest Specialist  (FL, FF, WB) | LC |
| 28 | Peregrine Falcon | *Falco peregrinus* | 0.01 | | 0.01 | 0.01 | | 0.01 | | 0.67 | R | Predator | Generalist  (UB, FF, FR) | LC |

Migratory Status (MS): R-Resident, W- Winter visitor, S-Summer visitor

Habitat Guilds: FR – Undisturbed forests; FF-Forest farmland interfaces; FL-Farmlands; UB-Urban built-up; GP-Green belt and urban avenue plantations; WB-Water bodies and buffer zones

IUCN: LC- Least Concern; NT-Near Threatened; VU- Vulnerable; EN- Endangered; CR- Critically Endangered
